# Predictors of short stature in Israeli children aged 6–7 years: a retrospective cohort study

**DOI:** 10.1186/s13584-025-00674-8

**Published:** 2025-03-05

**Authors:** Naama Fisch-Shvalb, Michal Yackobovitch-Gavan, Naomi Fliss-Isakov, Yair Morali, Nati Brooks, Moran Blaychfeld-Magnazi, Deena Rachel Zimmerman, Liora Lazar, Moshe Phillip, Ronit Endevelt

**Affiliations:** 1 Institute of Endocrinology and Diabetes, Schneider Children’s Medical Center, Petach Tikva, Israel; 2 Department of Pediatrics, Faculty of Medicine, Tel Aviv University, Tel Aviv, Israel; 3 Epidemiology and Preventive Medicine, School of Public Health, Faculty of Medicine, Tel Aviv University, Tel Aviv, Israel; 4 Nutrition Division, Public Health Directorate, Ministry of Health, Jerusalem, Israel; 5 Department of Health Promotion, School of Public Health, Faculty of Medicine, Tel-Aviv University, Tel Aviv, Israel; 6 Digital and Data Technologies Division, Israel Ministry of Health, Jerusalem, Israel; 7 Maternal Child and Adolescent Department, Public Health Directorate, Ministry of Health, Jerusalem, Israel; 8 School of Public Health, Haifa University, Haifa, Israel; 9 National Center for Childhood Diabetes, The Jesse Z and Sara Lea Shafer Institute for Endocrinology and Diabetes, Schneider Children’s Medical Center of Israel, 14 Kaplan St., 49202-35 Petah Tikva, Israel

**Keywords:** Short stature, Children, Height, Socioeconomic, Growth gap

## Abstract

There are differences in the rates of short stature (WHO height-z score < -2SD) between the various sectors in Israeli children aged 6–7 years, with higher rates in the ultraorthodox Jewish population. We aimed to: (a) Compare the anthropometric data at 0–2 years of age and the obstetric and demographic data of children with short stature at 6–7 years of age with those of children with normal height. (b) Assess risk factors for short stature at the age of 6–7 years. (c) Evaluate the impact of clinical and socioeconomic factors on linear growth from birth to the age of 6–7 years. This was a retrospective cohort study. Anonymized anthropometric data measured at the first grade of school during 2015–2019 were collected from the Ministry of Health records. The participants were stratified into sectors according to the affiliation of their school. Retrospective growth and sociodemographic data were extracted for each child from the national birth registry and Maternal Child Health Clinics files. The cohort included 368,088 children, with a median age of 6.7 years (IQR 6.3,7.0). Short stature was more prevalent in ultraorthodox Jewish boys (3.8%) and girls (3.2%), and least prevalent in Arab boys (0.8%) and girls (0.7%) compared with all other sectors (P < 0.001). The rate of stunting in Bedouin children was similar to that in the general population (1.6%). In a logistic regression model, the variables that predicted short stature at the age of 6–7 years were female sex, longer gestation, lower height z-score at 2 months of age, birth weight < 90th percentile, being in the ultraorthodox Jewish sector, and a smaller change in height z-score until 2 years of age. Growth gaps between different sectors of school-aged Israeli children emerge during the first 2 years of life. The most vulnerable population for stunting is the ultraorthodox population. Public health services, including Maternal Child Health clinics and primary caregivers, should prioritize this group and closely monitor for growth faltering during the first and second years of life.

## Background

Disparities in children's height are known to reflect socioeconomic inequalities and may predict health and developmental consequences in adulthood [1]. Extreme short stature is associated with an increased risk of morbidity and mortality and may be associated with cognitive development, educational performance, and less work productivity later in life [1, 2].

The Israeli Ministry of Health annually measures the height and weight of schoolchildren in the 1st and 7th grades, achieving excellent coverage rates (71–100%) during the years 2015–2019. Data from the Ministry of Health indicate that height and BMI indices vary among first graders (aged 6–7 years) across six different sectors in Israel: secular Jews, religious (orthodox) Jews, ultraorthodox Jews (UOJ), Arabs, Bedouins, and Druze. In 2021–2022, significant differences in short stature prevalence (height z-score (HAZ) < -2 SD according to WHO curves) were observed between the sectors (5% in UOJ versus 1–2% in other sectors) [3]. Among first graders, the proportion of children with short stature in the UOJ sector is twice the national average, and four times the rate among secular Jews. The cause of this disparity and the age at which it emerges remain unknown.

Infancy (ages 0–2 years) is a critical period of growth in childhood, as nutrition during the first 1,000 days of life has a significant and lasting impact on growth. A single study conducted in the United Kingdom compared the growth of infants in a UOJ community to that in the general population and reported that while UOJ children were born with weights similar to those of the rest of the cohort, they were significantly lighter and shorter by the age of one year, even after adjusting for parental height [4]. The authors attributed these growth patterns to larger family sizes and the delayed introduction of complementary foods, which are known risk factors for growth attenuation in less affluent societies [5, 6]. Early-life socioeconomic status can indeed affect linear growth [7]. The factors involved include maternal education [8, 9], factors related to pregnancy and childbirth [10], prolonged exclusive nursing beyond the recommended 6 months [11], quality and quantity of nutrition throughout childhood, food and nutrition security, and access to health services [7]. The rates of short stature and underweight among infants aged 0–2 years across the different sectors of Israeli society are still unknown.

Detailed data on the prevalence of short stature and the genetic, nutritional, and environmental factors contributing to it will aid in early diagnosis and intervention, improving health outcomes and quality of life for affected children. This information will also help public health officials and policymakers develop targeted strategies and healthcare policies to support children's growth and development, address disparities and promote overall health equity across various sectors of Israeli society.

## Methods

The aims of our study were as follows: (a) To compare the anthropometric data from early childhood (0–2 years) and the obstetric and demographic data of children with short stature at the age of 6–7 years (HAZ < -2) with those of children with a normal height. (b) To assess risk factors for short stature at the age of 6–7 years on the basis of growth and sociodemographic data at the age of 0–2 years. (c) To evaluate the impact of various clinical and socioeconomic factors on linear growth from birth to the age of 6–7 years.

## Design and setting

Well-child health records in Israel include regular anthropometric measurements for every child from birth to the age of six at the national maternal child health (MCH) clinics “Tipat Halav” [12] and during the 1st and 7th grades at school. This information is stored in the computerized database of the Israeli Ministry of Health (MoH).

In this retrospective cohort study, we extracted features and outcomes from three nationwide datasets:

1.Data from Machshava Briah, the electronic medical records of the MCH clinics [13]. There are approximately 1,000 MCH clinics distributed throughout the country, managed by the MoH, 2 municipalities (Tel Aviv and Jerusalem), or 1 of the 4 Israeli health maintenance organizations. This study is based on the database of visits to the clinics run by the MoH, the municipalities, and 1 health maintenance organization (Leumit), all of which use the same electronic medical record. This group of children represents approximately 70% of the Israeli children aged 0 to 6 years. The data included sex, length/height and weight measurements up to 2 years of age.

2.Data from the School Health electronic records included height and weight measurements taken routinely at school during the first grade, sector and socioeconomic position (SEP). Each school in Israel is classified as belonging to one of 6 sectors (secular Jew/religious Jew/UOJ/Arab/Bedouin/Druze), and each child in the cohort was assigned according to the affiliation of the educational institution the child attended.

3. Data from the national records of deliveries and childbirth, collected from all Israeli hospitals. The data included birth weight, gestational age at birth, maternal age and parity at delivery, number of siblings at current delivery.

The study protocol was approved by the MoH review board and was conducted in accordance with the principles of the Declaration of Helsinki. All the data were coded and anonymized and kept within the MoH, according to the institutional review board's instructions. An exemption from informed consent was granted by the ethics committee, as the data were anonymized.

## Population

The study population comprised all 1st grade students who underwent height and weight measurements by the MoH between the years 2015 and 2019, and had at least one anthropometric measurement from an MCH clinic between birth and 24 months. For each child, we then extracted weight and length data from ages 2, 6, 12, 18, and 24 months from MCH medical files as well as perinatal and sociodemographic data from the national birth registry. Short stature at 6–7 years of age was defined as a height for age-Z score (HAZ) < -2 SD according to WHO norms. **The inclusion criteria** were as follows: height and weight measurements from first grade, weight data at birth, and weight and length at 12 and 24 months of age. Children with outlier BMI-z scores (> 7 and < -6 SD) and children with missing data were excluded.

## Definitions

1. The SEP index determined by the Israel Central Bureau of Statistics is calculated using 14 variables measuring social and economic levels across demographics, education, standard of living, and employment. SEP is determined by home address, classifying towns and neighborhoods by their 2015 socioeconomic level. The SEP clusters, which are ranked from 1–10 (with 1 being the lowest), are categorized into three levels in the CHS database: low SEP (clusters 1–4), medium SEP (clusters 5–6), and high SEP (clusters 7–10) [14].
2. Birth weight was categorized according to sex and gestational age: appropriate for gestational age was defined as a weight for age between the 3rd and the 90th percentiles of Israeli newborns [15]. Small for gestational age (SGA) was defined as a weight < 3rd percentile for gestational age[16]. Large for gestational age (LGA) was defined as a weight > 90th percentile for gestational age.
3. BMI status categories at first grade were defined according to the WHO definitions [17]: underweight BMI-Z < -2, normal weight -2 ≤ BMI-Z < 1, overweight 1 ≤ BMI-Z ≤ 2, and obesity BMI-Z > 2.
4. Outlier z-score values were defined to a minimum (> 7 and < -6 SD), intended to exclude only potentially erroneous values [18].

## Statistical analysis

Statistical analyses were performed using SPSS software, version 29 (SPSS, Inc., Chicago, Illinois). Continuous variables with normal distributions are reported as means ± SDs, continuous variables with skewed distributions are reported as medians and interquartile ranges (IQRs), and categorical variables are reported as numbers and percentages. Comparisons of clinical and demographic variables between children with short stature (SS) and children with normal stature (NS, HAZ ≥ -2SD according to WHO norms) were performed via univariate analysis: independent sample t tests for continuous variables with a normal distribution, Mann‒Whitney U tests for continuous variables with a skewed distribution, and Pearson's chi‒square tests for categorical variables. Comparisons of clinical and demographic variables between different sectors were performed through one-way ANOVA and post hoc Tukey tests for continuous variables with a normal distribution, Kruskal‒Wallis tests and post hoc Nemenyi tests for continuous variables with a skewed distribution, and Pearson's chi‒square tests for categorical variables.

A logistic regression model was constructed to assess predictors of short stature at 6–7 years of age on the basis of growth characteristics in early childhood and obstetric and demographic data. Finally, linear repeated measures mixed models were constructed to evaluate the changes in HAZ scores over time from birth to 6 years. The models were specified with a within-group factor of time, a between-group factor (according to sex, stature category, SEP index category, BMI status category, sectors and birth weight for gestational age) and the interaction of group with time. In the mixed model analyses, the data are expressed as estimated marginal means and standard errors.

## Results

**Fig. 1:**
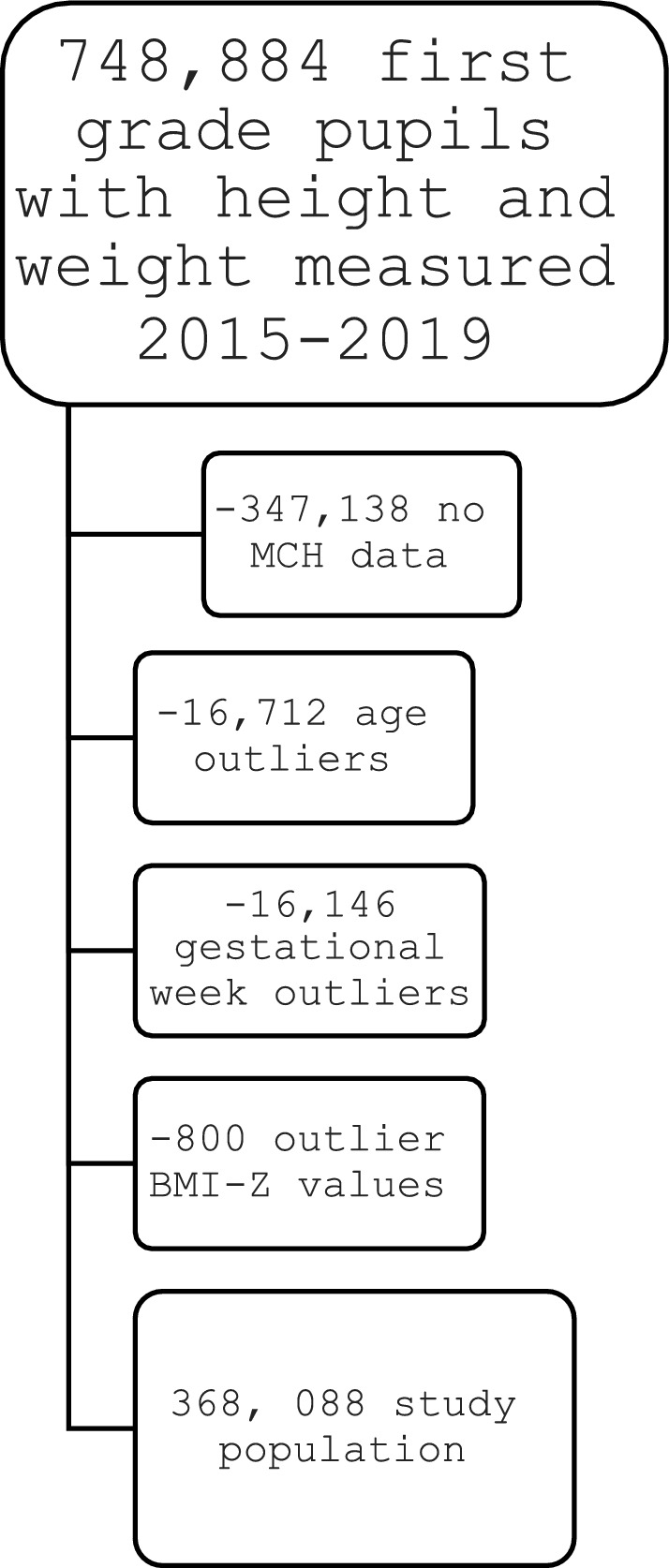
Study Flow Chart

The cohort comprised 368,088 first-grade pupils with sufficient data from 2015–2019 (Fig. 1), with a median age of 6.7 years (IQR 6.3–7.0). Two hundred thirty-three children had no data on sector (114 girls, 119 boys), leaving 367,855 children in the analyses according to sector.

**Fig. 2:**
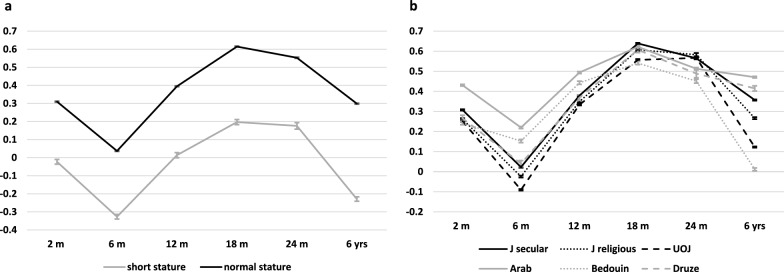
(pptx file): Title: BMI-Z from birth to age 6 years stratified according to: a. stature category, **b** sectors Legend: Short stature = HAZ < -2 SD according to WHO norms. UOJ = ultraorthodox Jewish

**Comparison of boys and girls with short stature to boys and girls with normal stature at 6–7 years (**Table 1**, **Fig. 2**): *Anthropometric data:*** The percentage of children with SS was 0.1% lower in girls than in boys (1.6% and 1.7%, respectively, P < 0.001). The median BMI-z of children with SS was lower than that of children with NS, both in boys and in girls (P < 0.001). The BMI-Z of short boys was lower than that of short girls (P < 0.001). According to a linear mixed model the BMI-Z of children with SS was lower than that of those with NS throughout the first 6 years of life (P_group_ < 0.001 & P_time×group_ < 0.001) (Fig. 2) but was within the normal range at all timepoints.

**Table 1:** A comparison between boys and girls with short stature and those with normal height, at 6–7 years

	Short stature n = 6145 (1.7%)	Normal height n = 361,943 (98.3%)	P1
Sex n (%) Male Female	3237 (1.7) 2908 (1.6)	181,875 (98.3) 180,068 (98.4)	< 0.001
Age, years Median (IQR) Male Female P2	6.7 (6.3, 7.0) 6.8 (6.5, 7.1) < 0.001	6.7 (6.3, 6.9) 6.7 (6.4, 7.0) < 0.001	0.758 < 0.001
HAZ age 6–7 Mean ± SD Male Female P2	-2.38 ± 0.36 -2.38 ± 0.38 0.894	0.12 ± 0.97 0.07 ± 0.94 < 0.001	< 0.001 < 0.001
BMI-z age 6–7 Median (IQR) Male Female P2	-0.33 (-0.83,0.21) -0.28 (-0.69,0.21) < 0.001	0.04 (-0.54,0.75) 0.09 (-0.45,0.80) < 0.001	< 0.001 < 0.001
Demographic data:			
SEP cluster Median (IQR) Male Female P2	2 [1, 5] 3 (1,6) 0.004	5 (2,7) 5 (2,7) 0.077	< 0.001 < 0.001
Number of siblings at birth Mean ± SD Male Female P2	1.06 ± 1.91) 0.94 ± 1.73 0.048	0.89 ± 1.50 0.88 ± 1.49 0.043	0.977 0.002
First child n (%) Male Female P2	2031 (62.7) 1884 (64.8) 0.096	110,026 (60.5) 108,457 (60.2) 0.104	0.009 < 0.001
Perinatal Data:			
Maternal age at birth, mean ± SD Male Female P2	29.0 ± 5.6 28.9 ± 5.7 0.410	29.3 ± 5.6 29.4 ± 5.6 0.006	0.004 < 0.001
Birth type n (%) Male *Vaginal* *CS* *instrumental* Female *Vaginal* *CS* *instrumental* P2	2467 (76.2) 612 (18.9) 158 (4.9) 2272 (78.1) 532 (18.3) 104 (3.6) 0.028	137,814 (75.8) 34,353 (18.9) 9708 (5.3) 140,921 (78.3) 31,684 (17.6) 7463 (4.1) < 0.001	0.515 0.219
Gestational age Median (IQR) Male Female P2	39 (38,40) 39 (38,40) 0.150	39 (38,40) 39 (38,40) < 0.001	0.004 0.003
Multiple fetus, n(%) Male Female P2	225 (7.0) 181 (6.2) 0.252	7946 (4.4) 8097 (4.5) 0.062	< 0.001 < 0.001
Corrected Birth weight n(%) Males AGA SGA LGA Females AGA SGA LGA P2	2965 (91.6) 185 (5.7) 87 (2.7) 2676 (92.0) 175 (6.0) 57 (2.0) 0.155	161,044 (88.5) 2532 (1.4) 18,299 (10.1) 159,373 (88.5) 2524 (1.4) 18,171 (10.1) 0.925	< 0.001 < 0.001

Continuous variables with normal distributions were reported as means ± SD, continuous variables with skewed distributions were reported as medians and inter-quartile ranges (IQR), and categorical variables were reported by numbers and percentages

P1 represents the comparison between children with short stature and normal height, stratified by sex. P2 represents the comparison between males and females within each stature category. Independent sample t-tests were used for continuous variables with normal distribution, Mann–Whitney U tests were used for continuous variables with skewed distribution, and Pearson's chi-square tests were used for categorical variables

HAZ = height z-score; SEP = socioeconomic position; CS = cesarean section; AGA = appropriate for gestational age; SGA = small for gestational age; LGA = large for gestational age

### Demographic data

The SEP of both boys and girls with SS was lower than that of children with NS (boys: median cluster 2 (IQR 1,5) vs. 5 (IQR 2,7); girls: median cluster 3 (IQR 1,6) vs. 5 (IQR 2,7), P < 0.001 for both). Within the short stature group, boys had a lower median SEP index than girls (2 (IQR 1,5) vs. 3 (IQR 1,6), P < 0.001). A greater percentage of children with SS were first children in the family than were children with NS (boys: P = 0.009; girls: P < 0.001).

### Perinatal data

The mean maternal age at birth for children with SS was several months younger than that for children with NS for both boys and girls (P = 0.004 and P < 0.001, respectively). A greater percentage of children in the SS group, both boys and girls, were born in multiple pregnancies (P < 0.001). Compared with children with NS, more children born SGA were in the SS group (boys: 5.7% vs. 1.4%, girls: 6.0% vs. 1.4%, P < 0.001 for both), and fewer children were born LGA (boys: 2.7% vs. 10.1%, P < 0.001; girls: 2.0% vs. 10.1%, P < 0.001).

## Characteristics of the cohort according to sector (Table 2, Fig. 2):

**Table 2:** Between sector comparisons for boys and girls at age 6–7 years

	Jewish secular	Jewish orthodox	Jewish ultra−orthodox	Arab	Bedouin	Druze	P1	
Total n (% of cohort) Male Female	70,769 (38.3) 69,853 (38.2)	26,147 (14.1) 26,604 (14.5)	44,143 (23.9) 43,366 (23.7)	30,562 (16.5) 30,250 (16.5)	9985 (5.4) 9557 (5.2)	3387 (1.8) 3232 (1.8)		
Short stature n (% within sector) Male Female P2	655 ^a^ (0.9) 635 ^a^ (0.9) 0.767	484 ^b^ (1.9) 476 ^b^ (1.8) 0.618	1661 ^c^ (3.8) 1406 ^c^ (3.2) < 0.001	243 ^d^ (0.8) 222 ^d^ (0.7) 0.412	165 ^b^ (1.7) 133 ^e^ (1.4) 0.153	29^a,d^ (0.9) 33^a,d,e^ (1.0) 0.570	< 0.001 < 0.001	
HAZ mean ± SD Male Female P2	0.24 ^a^ ± 1.00 0.20 ^a^ ± 0.97 < 0.001	0.00 ^b^ ± 1.00 −0.06 ^b^ ± 0.97 < 0.001	−0.31 ^c^ ± 0.98 −0.32 ^c^ ± 0.95 0.123	0.32 ^d^ ± 0.98 0.26 ^d^ ± 0.95 < 0.001	−0.02 ^b^ ± 0.97 −0.06 ^b^ ± 0.93 0.009	0.20^e^ ± 0.93 0.12^e^ ± 0.95 < 0.001	< 0.001 < 0.001	
BMI−z median (IQR) Male Female P2	0.07 ^a^ (−0.53,0.84) 0.14 ^a^ (−0.43,0.90) < 0.001	−0.03 ^b^ (−0.58,0.68) 0.09 ^b^ (−0.45,0.79) < 0.001	−0.08 ^c^ (−0.60,0.52) 0.01 ^c^ (−0.47,0.61) < 0.001	0.23^d^ (−0.37, 0.94) 0.20 ^d^ (−0.39, 0.91) 0.144	−0.16 ^e^ (−0.70, 0.47) −0.17 ^e^ (−0.67, 0.46) 0.392	0.19^d^ (−0.44, 0.87) 0.13^a^ (−0.42, 0.84) 0.413	< 0.001 < 0.001	
SEP median (IQR) Male Female P2	7(6,8)^a^ 7(6,8)^a^ 0.707	5 (4,7)^b^ 5 (4,7)^b^ 0.067	2 (1,3)^c^ 2 (1,3) ^c^ < 0.001	3 (2,3)^d^ 3 (2,3)^d^ 0.001	1 (1,2)^e^ 1 (1,2)^e^ 0.106	4 (3,5)^f^ 4 (3,5) ^f^ 0.865	< 0.001 < 0.001	
Maternal age (years) Mean ± SD Male Female P2	31.3 ± 4.8 ^a^ 31.4 ± 4.9 ^a^ < 0.001	29.3 ± 5.4 ^b^ 29.4 ± 5.4 ^b^ 0.041	28.2 ± 5.8 ^c^ 28.2 ± 5.8 ^c^ 0.224	27.2 ± 5.5^d^ 27.1 ± 5.5^d^ 0.277	27.3 ± 5.9^d^ 27.4 ± 5.9^e^ 0.437	27.7 ± 5.2^e^ 27.9 ± 5.3 ^f^ 0.168	< 0.001 < 0.001	
Birth type % Male * Vaginal* * CS* * Instrumental* Female * Vaginal* * CS* * Instrumental* P2	67.7 ^a^ 25.7 6.6 70.7 ^a^ 23.9 5.4 < 0.001	76.3 ^b^ 17.8 5.9 79.0 ^b^ 16.4 4.5 < 0.001	84.4 ^c^ 11.2 4.4 86.2 ^c^ 10.5 3.3 < 0.001	80.1 ^d^ 16.2 3.7 82.3 ^d^ 15.0 2.7 < 0.001	80.6^d^ 15.5 3.9 82.1^d^ 15.1 2.8 < 0.001	74.2 ^e^ 21.1 4.8 79.1^b^ 18.2 2.8 < 0.001	< 0.001 < 0.001	
Gestational age, weeks Male Female P2	39(38,40) ^a^ 39(38,40) ^a^ < 0.001	39(38,40) ^b^ 39(38,40) ^b^ < 0.001	40(39,40) ^c^ 40(39,41) ^c^ < 0.001	39(38,40) ^d^ 39(38,40) ^d^ < 0.001	39(38,40)^b^ 38(38,40)^b^ 0.039	39(38,40) ^b^ 39(38,40) ^b^ 0.279	< 0.001 < 0.001	
Multiple fetus% Male Female P2	5.4 ^a^ 5.7 ^a^ 0.069	4.4 ^b^ 4.4 ^b^ 0.998	3.5 ^c^ 3.4 ^c^ 0.577	4.0 ^d^ 4.1^b^ 0.348	3.1^e^ 3.3^c^ 0.316	3.2^e^ 3.2^c^ 0.994	< 0.001 < 0.001	
Birth Wt for GA n(%) Male LGA SGA Female LGA SGA P2	6783(9.6) ^a^ 977(1.4) ^a^ 6724(9.6) ^a^ 956(1.4) ^a^ 0.790	2450(9.4) ^a^ 408(1.6) ^a^ 2504(9.4) ^a^ 426(1.6) ^b^ 0.902	4666(10.6)^b^ 617(1.4)^a^ 4590(10.6)^b^ 655(1.5)^ab^ 0.376	3354(11.0)^b^ 421(1.4)^a^ 3266(10.8)^b^ 409(1.4)^a^ 0.746	808(8.1)^c^ 215(2.2)^b^ 821(8.6)^c^ 210(2.2)^c^ 0.436	308(9.1)^a^ 58(1.7)^ab^ 303(9.4)^ac^ 40(1.2)^ab^ 0.246	< 0.001 < 0.001	
First child% Male Female P2	58.0 ^a^ 57.9 ^a^ 0.922	58.4 ^b^ 57.2 ^a^ 0.007	69.2^c^ 69.5^b^ 0.487	55.7^d^ 55.5^c^ 0.722	68.4^e^ 67.8^b^ 0.402	38.8^f^ 36.9^d^ 0.120	< 0.001 < 0.001	

P1 represents Comparisons between sectors using one-way ANOVA and post hoc Tukey test for continuous variables with normal distribution, Kruskal–Wallis tests and post hoc Nemenyi test for continuous variables with skewed distribution, and Pearson's chi-square tests for categorical variables. Rates with different superscripts (a, b, c, d, e, f) differ significantly from each (P < 0.05). P2 represents the comparison between males and females within each sector category using independent sample t-tests for continuous variables with normal distribution, Mann–Whitney U tests for continuous variables with skewed distribution, and Pearson's chi-square tests for categorical variables

HAZ = height z-score; SEP = socioeconomic position; CS = cesarean section; AGA = appropriate for gestational age; SGA = small for gestational age; LGA = large for gestational age

All between-sector comparisons were significant (P < 0.001).

***Anthropometric data:*** Short stature at the age of 6–7 years was most prevalent in UOJ boys (3.8%) and girls (3.2%) and least prevalent in Arab children (boys, 0.8%; girls, 0.7%). The mean HAZ was also lowest in the UOJ boys (-0.31 ± 0.98) and girls (-0.32 ± 0.95) and highest in the Arab boys (0.32 ± 0.98) and girls (0.26 ± 0.95). Among children with SS, 46.9% of girls and 50.2% of boys were from the UOJ sector, although UOJ girls comprised only 23.7% and UOJ boys only 23.9% of the overall cohort. The median BMI-z at 6–7 years was lowest among Bedouin boys and girls and highest among Arab boys and girls and Druze boys. According to a linear mixed model, BMI-Z throughout childhood differed between sectors (P_group_ < 0.001 & P_time×group_ < 0.001 (Fig. 2) and between time points (P_time_ < 0.001) but remained within normal range at all time points and across all sectors.

***Demographic data*:** The median SEP index was the lowest in the Bedouin sector (1 (IQR 1,2)) and in the UOJ sector (2 (IQR 1,3)). The highest median SEP index was found among secular Jews (7 (IQR 6,8)). The mean maternal age at birth was highest among secular Jewish children (boys 31.3 ± 4.8 years, girls 31.4 ± 4.9) and lowest among Arab children (boys 27.2 ± 5.5, girls 27.1 ± 5.5) and Bedouin boys (27.3 ± 5.9 years). ***Perinatal data:*** Compared with all other sectors, the mean maternal age at birth was highest among secular Jewish children and lowest among Arab children and Bedouin boys. The median gestational age at birth was highest among UOJ children. The normal delivery rate was lowest among secular Jewish children, who also had the highest C-section rate; UOJ children had the lowest C-section rate. The highest percentage of LGA births was reported among UOJ and Arab children, whereas the lowest percentage was reported among Bedouin children. The percentage of children born SGA was highest among Bedouin children.

## Predictive models of short stature at the age of 6–7 years (Table 3):

**Table 3:** Logistic regression models for factors predicting short stature (HAZ < -2.0 SDS) at age 6–7 years

	0R (95% CI)	P value
**Demographic**		
Male sex	0.747 (0.664, 0.840)	** < 0.001**
First child	1.080 (0.952, 1.226)	0.223
SEP cluster	1.017(0.983, 1.051)	0.334
Sector Jewish secular (Reference) Jewish orthodox Jewish ultra-orthodox Arab Bedouin Druze	1.00 1.103 (0.899, 1.354) 1.598 (1.295, 1.973) 0.996 (0.781, 1.271) 0.854 (0.633, 1.151) 1.520 (0.864, 2.672)	0.346 ** < 0.001** 0.977 0.299 0.148
**Perinatal**		
Gestational age	1.117 (1.079, 1.157)	** < 0.001**
Maternal age	1.001 (0.990, 1.012)	0.812
Multiple fetus	1.037 (0.795, 1.352)	0.788
LGA	0.509 (0.319, 0.810)	**0.004**
SGA	1.028 (0.791, 1.336)	0.837
**Anthropometric**		
HAZ 2 months	0.082 (0.075, 0.089)	** < 0.001**
Delta HAZ 2–6 months	0.076 (0.069, 0.084)	** < 0.001**
Delta HAZ 6–12 months	0.086 (0.076, 0.096)	** < 0.001**
Delta HAZ 12–18 months	0.118 (0.104, 0.134)	** < 0.001**
Delta HAZ 18–24 months	0.246 (0.219, 0.277)	** < 0.001**

HAZ = height z-score; SEP = socioeconomic position; SGA = small for gestational age; LGA = large for gestational age

In a logistic regression model, the following characteristics were predictive of having short stature at the age of 6–7 years: UOJ sector, advanced gestational age, a lower HAZ at 2 months of age, and a lower Δ HAZ in the first 24 months of life. The effect of a lower Δ HAZ was strongest in the first six months of life and gradually diminished until the age of two. The protective factors against having short stature at the age of 6–7 years were male sex and birth weight above the 90th percentile for gestational age (LGA).

## Overtime trajectories of linear growth from birth to the age of 6–7 years according to various clinical and socioeconomic factors (Table 4 and Fig. 3)

**Table 4:** Height-Z from birth to age 6–7 years stratified according to possible confounders

(n)	0–4 months	6 months	12 months	18 months	24 months	6 years	P-time	P-group	P time × group
Boys (185,112) Girls (182,976)	0.096(0.007)^a^ 0.083(0.006)^a^	0.023(0.007)^b^ 0.062(0.006)^b^	-0.164(0.007)^c^ -0.130(0.006)^c^	-0.321(0.007)^d^ -0.253(0.006)^d^	-0.354(0.007)^e^ -0.289(0.007)^e^	-0.173(0.007)^f^ -0.198(0.006)^f^	< 0.001 < 0.001	0.251	< 0.001
Short stature (6145) Normal height (361,943)	-1.088(0.013)^a^ 0.351(0.002)^a^	-1.495(0.013)^b^ 0.310(0.002)^b^	-1.907(0.014)^c^ 0.133(0.002)^c^	-2.090(0.014)^d^ -0.004(0.002)^d^	-2.121(0.018)^d^ -0.055(0.003)^e^	-2.376(0.012)^e^ 0.095(0.002)^f^	< 0.001 < 0.001	< 0.001	< 0.001
High SEP (8–10) (110,125) Medium SEP (4–7) (78,266) Low SEP (1–3) (167,431)	0.404(0.004)^a^ 0.346(0.004)^a^ 0.269(0.003)^a^	0.435(0.004)^b^ 0.374(0.004)^b^ 0.138(0.003)^b^	0.239(0.004)^c^ 0.193(0.005)^c^ -0.040(0.003)^c^	0.091(0.004)^d^ 0.037(0.005)^d^ -0.163(0.003)^d^	0.022(0.006)^e^ -0.007(0.007)^e^ -0.194(0.004)^d^	0.179(0.003)^f^ 0.114(0.004)^f^ -0.057(0.003)^e^	< 0.001 < 0.001 < 0.001	< 0.001	< 0.001
Underweight (723) Normal weight (295,992) Overweight (41,039) Obese (30,300)	-0.390(0.052)^a^ 0.286(0.002)^a^ 0.484(0.006)^a^ 0.536(0.007)^ab^	-0.406(0.053)^a^ 0.220(0.002)^b^ 0.482(0.006)^a^ 0.614(0.007)^b^	-0.518(0.054)^b^ 0.024(0.002)^c^ 0.345(0.006)^b^ 0.519(0.007)^a^	-0.700(0.055)^c^ -0.117(0.002)^d^ 0.214(0.006)^c^ 0.394(0.007)^c^	-0.761(0.072)^c^ -0.182(0.003)^e^ 0.193(0.009)^c^ 0.390(0.010)^c^	-0.327(0.049)^a^ -0.078(0.002)^f^ 0.405(0.006)^d^ 0.877(0.006)^d^	< 0.001 < 0.001 < 0.001 < 0.001	< 0.001	< 0.001
J secular (140,622) J orthodox (52,751) UOJ (87,509) Arab (60,812) Bedouin (19,542) Druze (6619)	0.397(0.003)^a^ 0.299(0.005)^a^ 0.265(0.004)^a^ 0.328(0.005)^a^ 0.074(0.009)^a^ 0.644(0.015)^a^	0.472(0.003)^b^ 0.197(0.005)^b^ 0.000(0.004)^b^ 0.356(0.005)^b^ -0.026(0.009)^b^ 0.641(0.015)^a^	0.293(0.003)^c^ -0.032(0.006)^c^ -0.301(0.004)^c^ 0.302(0.005)^c^ -0.136(0.009)^c^ 0.436(0.014)^b^	0.130(0.003)^d^ -0.162(0.006)^d^ -0.400(0.004)^d^ 0.160(0.005)^d^ -0.268(0.009)^d^ 0.210(0.014)^c^	0.072(0.005)^e^ -0.215(0.009)^e^ -0.446(0.006)^e^ 0.138(0.006)^e^ -0.259(0.011)^d^ 0.140(0.021)^d^	0.219(0.003)^f^ -0.028(0.005)^c^ -0.315(0.004)^f^ 0.291(0.005)^c^ -0.039(0.008)^b^ 0.162(0.013)^d^	< 0.001 < 0.001 < 0.001 < 0.001 < 0.001 < 0.001	< 0.001	< 0.001
SGA (5416) AGA (323,058) LGA (36,614)	-1.185(0.018)^a^ 0.241(0.002)^a^ 1.316(0.006)^a^	-0.838(0.018)^b^ 0.216(0.002)^b^ 1.023(0.006)^b^	-0.783(0.018)^c^ 0.045(0.002)^c^ 0.735(0.006)^c^	-0.890(0.018)^d^ -0.087(0.002)^d^ 0.533(0.006)^d^	-0.869(0.025)^bd^ -0.140(0.003)^e^ 0.459(0.009)^e^	-0.534(0.017)^e^ 0.012(0.002)^f^ 0.517(0.006)^d^	< 0.001 < 0.001 < 0.001	< 0.001	< 0.001

Linear repeated measures mixed model for the change in height-Z score from birth to 6 years. Data is presented as estimated mean and standard error (SE). Rates with different superscripts (a, b, c, d, e, f) differ significantly from each other (P < 0.05)

HAZ = height z-score; SEP = socioeconomic position; AGA = appropriate for gestational age; SGA = small for gestational age; LGA = large for gestational age

Underweight BMI-Z < -2SD, normal weight -2SD ≤ BMI-Z < 1SD, overweight 1SD ≤ BMI-Z ≤ 2SD, Obesity BMI-Z > 2SD according to WHO norms

**Fig. 3:**
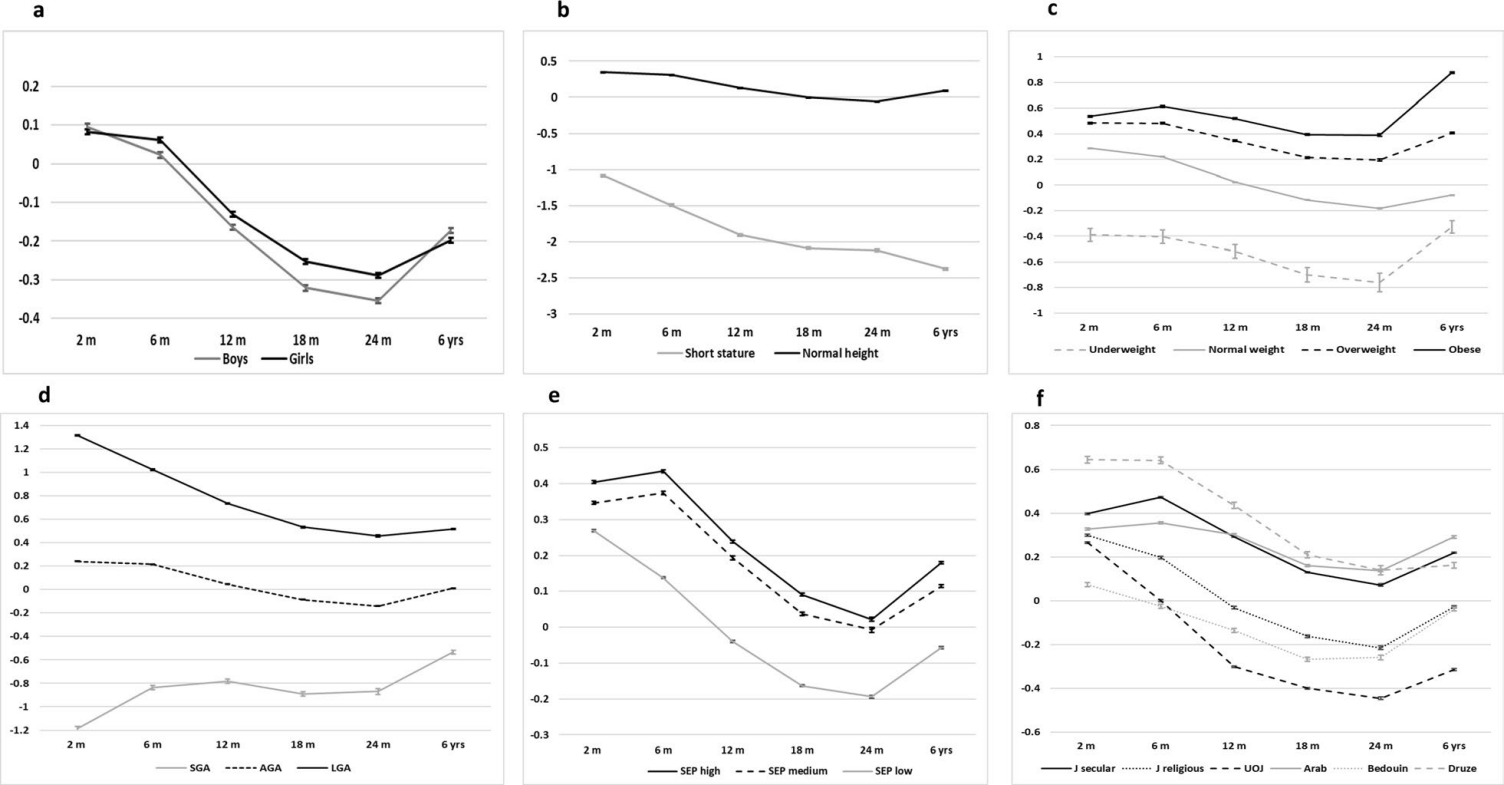
Height-Z trajectories from birth to age 6 years according to clinical and socioeconomic factors. Legend: Height-Z from birth to age 6 years stratified according to: a sex, b stature category, c BMI status category. d corrected birth weight category. e. socioeconomic category. f. sector. Short stature = height-z < -2 SD. BMI categories: Underweight BMI-Z < -2SD, normal weight-2SD ≤ BMI-Z < 1SD, overweight 1SD ≤ BMI-Z ≤ 2SD, Obesity BMI-Z > 2SD according to WHO norms. AGA, appropriate for gestational age; SGA, small for gestational age; LGA, large for gestational age. SEP, Sociodemographic position. OUJ, ultraorthodox Jewish

In the repeated measures linear mixed models, children in all subcategories exhibited significant changes in HAZ over time (P_time_ < 0.001, Fig. 3 a-f). Both boys and girls presented similar average values over time in the HAZ (P_group_ = 0.251), but their growth patterns diverged, with boys generally being taller by the age of 6 years (P_time×group_ < 0.001) (Fig. 3a). In both sex groups, HAZ decreased between time points, reaching a nadir at 2 years of age, followed by an increase in HAZ by the age of 6 years.

***Anthropometric factors:*** Children with SS at the age of 6 years were shorter than those with NS from the initial measurement, showing a continued decline until 24 months of age and no improvement in HAZ until the age of 6 years, unlike children with NS (P_group_ < 0.001 & P_time×group_ < 0.001) (Fig. 3b). The mean difference in HAZ scores between short children and those with NS was 1.44 SD at 2 months and increased to 2.47 SD by the age of 6 years.

When the data were subcategorized according to BMI-z at 6 years, a divergence in HAZ between subgroups was evident from 2 months of age (Fig. 3c). HAZ scores increased between 24 months and 6 years of age, particularly among obese and underweight children (P_group_ < 0.001 & P_time×group_ < 0.001).

When classified according to BW for gestational age (Fig. 3d), infants born LGA presented a pattern of catch-down growth until the age of 2 years, whereas those who were born SGA presented partial catch-up growth until the age of 2 years. Both groups further improved their HAZ between the ages of 2 and 6 years (P_group_ < 0.001 & P_time×group_ < 0.001). By age 6, the mean difference in HAZ between children born LGA and those born SGA was 1.051 SD.

***Socioeconomic factors:*** Children from higher SEP categories presented consistent and greater growth trends, maintaining above-average HAZs. Conversely, children from lower SEP categories presented a lower HAZ, with more pronounced decreases over time (P_group_ < 0.001 & P_time×group_ < 0.001). However, all categories improved between the ages of 24 months and 6 years (Fig. 3e). By age 6, the mean difference in the HAZ between high- and low-SEPs was only 0.236 SD.

The growth trends among children in different sectors varied substantially (Fig. 3f). While children in all sectors had a normal HAZ at 2 months of age, which decreased only from 6 months to a nadir at 2 years, the HAZ of UOJ started to decline at 2 months. The HAZ of OUJ children declined by 0.566 SD within the first year of life alone. At the age of 6 years, the difference between the tallest sector (Arabs) and the shortest sector (UOJ) was 0.606 SD (P_group_ < 0.001 & P_time×group_ < 0.001).

## Discussion

While Israel is a developed, industrialized country with free comprehensive healthcare services provided by a national health insurance law, it is also a country with high fertility rates, large socioeconomic gaps and the highest level of child poverty among OECD countries [19]. The aim of this study was to describe the growth trajectories of Israeli children from birth to the age of 6 years. We assessed the rate of short stature at 6 years, looked for disparities in linear growth to highlight children who are at risk for stunting, and aimed to identify a potential window for intervention to prevent stunting.

The rate of short stature at age 6 years in this cohort was low, at 1.6%, whereas the rest of the cohort had normal height compliant with WHO norms. Boys and girls presented similar trends overall, but the girls had a greater HAZ than the boys did up to the age of two. By the age of 6, the boys caught up, and their HAZ was greater than the girls' HAZ. Interestingly, a recent meta-analysis on sex differences in childhood growth under conditions of undernutrition [20] revealed that infant boys have greater odds of growth faltering in the context of undernutrition than infant girls do; however, this difference disappears after the age of 30 months. This course is also evident in our cohort.

In this study, children with short stature at age 6 are a heterogeneous group characterized by a lower BMI-Z at age 6, a low median SEP, a high rate of SGA and a low rate of LGA births compared to the general population. Most of these children belong to the UOJ sector. Their HAZ was within norms at 2 months of age (mean HAZ -1.0 SD), progressively declining to a mean of -2.38 SD at the age of 6 years. This implies a substantial postnatal contribution to their growth failure. Children with short stature continued to fall behind in HAZ between ages 2 and 6 years (-0.25 SD), while all other subgroups improved their HAZ during this period, suggesting continued exposure to the cause of their growth failure or failure to catch up from the growth insult.

In our cohort, children from the UOJ sector are shorter at age 6 than their peers despite being born with higher rates of LGA (a protective parameter according to our prediction model), having a longer mean gestation, having fewer multiple pregnancies and a normal mean HAZ at 2 months (0.26 SD). These data reflect normal growth during the prenatal period and point out to later growth faltering in this population. The steepest decline in the mean HAZ in UOJ infants occurred during the first year of life (Fig. 3f). This pattern is highly suggestive of stunting related to SEP and undernutrition [21]. SEP has been previously linked to stunting in children, both in developing and in industrialized countries, due to poorer diet quality and quantity, and poorer access, compliance to- or lack of education regarding health promoting behaviors (7–11, [22]. Specifically, poor dietary diversity and low consumption of animal-sourced foods in children from low SEP are inversely related with infant growth [23, 24]. In contrast, a diet rich in animal-source protein has been shown to promote linear growth and catch-up growth in children previously exposed to undernutrition [25, 26]. It is worth noting that poor dietary diversity is associated with stunting, but not with wasting [24], a pattern which was found in the UOJ population in our study.

The UOJ sector in Israel exhibits distinct socio-demographic and cultural characteristics that differentiate it from other sectors. According to the Israeli Bureau of Statistics [27], from 2015–2019, 36–39% of the UOJ families lived in poverty, compared to 21–23% of the families in the general Israeli population. Possible explanations for the higher rate of stunting in UOJ children include inadequate nutrition [19], longer duration of breastfeeding [4], and large families [28, 29]. Furthermore, the OUJ population may have different conceptual beliefs regarding the importance of healthy eating and preventive medicine. As Peles et al. concluded from interviews with OUJ opinion-leaders on their perspectives on healthy nutrition [30], spiritual perceptions regarding food, such as “Over preoccupation with healthy food is perceived as worship of the body” and “Success may be more likely if greater emphasis is placed on spiritual benefits and learning, rather than long-term physical health”, may influence the dietary habits of OUJ families and children. Finally, less exposure by choice to different media channels could lead to decreased awareness of the need of growth surveillance in children, as has been shown in other areas of healthcare in this population [31]. Unfortunately, as we do not have data regarding family size, the extent and duration of breastfeeding, or nutritional parameters during the first two years of life in our cohort, we cannot determine the cause of short stature in this sector based on our data.

We were surprised to find that Bedouin children, whose mean SEP index and BMI-Zs were the lowest compared to all sectors, did not have a higher rate of stunting at the age of 6, than did the general population, whereas UOJ children did. Stunting in Bedouin children was highly prevalent several decades ago, with a marked improvement between the years 2000–2010 [32]. This shift may be attributed to an improvement in maternal education [19], an increase in urban residence, or changes in diet [32]. This could also imply the success of national programs aimed at improving child healthcare in this population. Our current data revealed a further reduction in the stunting rate in Bedouin children to a rate that is comparable with the national mean. If we could unravel how linear growth has improved in this population, this could help us implement lessons learned in other populations who are at risk for stunting.

**Strengths and limitations:** To the best of our knowledge, this is the only contemporary longitudinal analysis of the growth trajectories of Israeli children to date. We utilized national registries to create a comprehensive and robust database including a large proportion of the Israeli population. Data was systematically gathered using the same methodology over years, in electronic health records which were routinely monitored. We further used stringent exclusion criteria to minimize missing data and reduce information bias.

Regrettably, we have no data on parental height, although genetic background should be similar across Jewish sectors [33]. The Jewish population in Israel, whether classified as Ashkenazi (European) or non-Ashkenazi (according to the country of origin), consists of a shared Near East ancestry regardless of their religious tendencies. Neither do we have data on parental education, an important determinant of childhood growth [9]. We can estimate the rates of maternal education according to national data from 2018 [34]: 80.6% of the non-UOJ-Jewish students completed 12 years of education, compared with 31% of the OUJ women, 64.8% of the Arab students, 82.2% of the Druze students and 52.1% of the Bedouin students.

Furthermore, we have no data on family size in this cohort. Again, we can only provide statistical data on the number of children in each of the sectors evaluated in this study. In 2015, the average number of children in an UOJ family was 6.6, whereas it was 2.2 for secular Jewish families and 4.2 for religious Jewish families. The average number of children per Arab family was 3.3; in Bedouin families, it was 5.5; and in Druze families, it was 2.2 children per family [35]. According to various studies, there is a negative impact of family size on children's height [36], with a decrease in final height of up to 2 cm in children with 9 or more siblings compared with those brought up as an only child [28]. The mechanism behind the effect of family size on height, whether nutritional, infectious or other environmental, is unknown. In our cohort, the OUJ and Bedouins both have large families and low SEP, yet one sector has a much higher rate of stunting than the other does, and the reasons for this observation remain to be determined.

Despite excellent mean coverage of school height and weight measurements—96.4% of all schools—the rate of coverage was lower in some sectors (85.8% in Druze schools, 88.2% in Bedouin schools, and 94.9% in UOJ schools). Furthermore, we have no data on children who are not enrolled in the public school system, or children who did not visit one of the MCH clinics from which data could be retrieved. We have no data on the organic causes of short stature or the rates of referral of children with short stature to receive medical evaluation and treatment across the different sectors.

## Conclusions and implementation

In this study, we found differences in the rates of stunting in Israeli children, which are not present at birth, but emerge mainly during the first two years of life and persist at least until the age of 6 years. This marks a clear window of opportunity for preventive interventions among population groups who are at higher risk for short stature, particularly the UOJ sector.

Early detection of nutritional insecurity or growth failure in high-risk infants could be facilitated by the MCH clinics. Within these clinics, necessary measures for prevention of lifestyle related stunting should be promoted with parental education directed at breastfeeding and a timely introduction of complementary feeding. A national program providing breastfeeding guidance and healthy food for mothers to feed their infants in low income societies, such as the WIC program in USA, has been shown to improve infant growth and nutritional intake [37, 38], and should be considered by Israeli decision makers.

Although our study points to growth faltering in the first 2 years of life, nutritional interventions delivered beyond this age can also promote catch-up growth in children previously exposed to suboptimal nutrition [39]. Governmental funding, guidance and provision of meals in early life education systems have been shown to improve the nutritional value and quality of meals provided in the UK and in Australia [40, 41]. In Israel, there are national guidelines for nutrition in child-care systems under government supervision, and these guidelines should be more strictly observed. Unfortunately, many children between 3 months and 3 years of age in Israel study in systems which are not supervised, and may not provide the necessary nutritional standard for proper growth and development. Work is underway to promote regulation and adherence to the recommended guidelines.

In conclusion, we strongly recommend that public health services, including MCH clinics and primary caregivers, prioritize the UOJ sector and other populations of lower socio-economic status, in screening for childhood stunting particularly during the first and second years of life. Interventions to prevent stunting should include health education, and perhaps also the provision of supplemental food in societies prone to nutritional insecurity. Strengthening this important preventive service for maternal and child health is an important means of promoting equity and providing all Israeli children with an equal opportunity for optimal growth.

## Data Availability

The data that support the findings of this study are available from the Ministry of Health, but restrictions apply to the availability of these data, which were used under license for the current study and so are not publicly available.

## Abbreviations

BMI: Body mass index
WHO: World health organization
HAZ: Height-z score
MCH: Maternal child health
UOJ: Ultraorthodox
SEP: Socioeconomic position
SS: Short stature
NS: Normal stature
SGA: Small for gestational age
LGA: Large for gestational age
